# Making tracks: microglia and the extracellular matrix

**DOI:** 10.1186/s13024-025-00898-x

**Published:** 2025-09-29

**Authors:** Lauren K. Wareham, David J. Calkins

**Affiliations:** Department of Ophthalmology and Visual Sciences, Vanderbilt Eye Institute, Vanderbilt University Medical Center, Nashville, TN 37212 USA

**Keywords:** Extracellular matrix, Microglia, Neurodegeneration, Cell motility, Mechanotransduction, Biomechanics, Blood-brain barrier

## Abstract

Microglia are resident immune cells of the central nervous system (CNS) and critical regulators of neural homeostasis, mediating immune surveillance, synaptic remodeling, debris clearance, and inflammatory signaling. Emerging evidence highlights the extracellular matrix (ECM) as important to microglial behavior in both physiological and pathological contexts. The CNS ECM is a dynamic and bioactive scaffold composed of three primary compartments: interstitial matrix, basement membranes at neurovascular and neuroepithelial interfaces, and perineuronal nets (PNNs). Each compartment exhibits distinct molecular architectures, ranging from fibrillar collagens and glycoproteins in basement membranes to chondroitin sulfate proteoglycans and hyaluronan-rich structures in PNNs. In this review we examine how microglia engage with and reshape the ECM to dynamically respond to disruptions in homeostasis with aging and disease. We discuss the concept of the microglial–ECM “interactome”, which may represent a molecular interface through which microglia sense, modify, and respond to their extracellular environment. This interactome enables microglia to enact fine-scale ECM remodeling during routine surveillance, as well as large-scale alterations under pathological conditions to help preserve function and motility. In aging and disease, dysregulation of the microglial-ECM interactome is characterized by aberrant mechanotransduction, elevated proteinase activity, remodeling of the ECM, and sustained pro-inflammatory cytokine release. These pathological changes compromise ECM integrity, challenge microglial activity, and contribute to progressive neurovascular and synaptic dysfunction. Deciphering the molecular mechanisms underpinning microglial–ECM interactions is essential for understanding region-specific vulnerability in neurodegeneration and may reveal new therapeutic targets for preserving ECM structure and countering CNS disorders.

## Background

Microglia, astrocytes and oligodendrocytes comprise the family of cells in the central nervous system (CNS) known historically as ***neuroglia***, so named because of their early depictions as adhesive connectors between neurons [1, 2]. While understanding of their rich and diverse functional contributions to the CNS has improved vastly, the name for these cells (or at least the glia part of it) has stuck, so to speak. Since their recognition as a distinct cell type just over a century ago [3], for several reasons, microglia have stood out among the three, while astrocytes and oligodendrocytes comprise what we call ***macroglia***. Microglia are morphologically complex and multifaceted glia cells with a broad functional repertoire. Microglia are the innate immune cells of the CNS, with important roles in classical immune surveillance and response to insult or disease. Microglia actively survey for danger-associated molecular patterns (DAMPs), e.g., cells undergoing apoptosis, accumulations of cytotoxic or neurotoxic signals, or reactive oxygen species [4].

As resident macrophages in the CNS microglia are behind the blood-brain barrier helping detect injury, cellular debris, and invasive pathogens within the neuronal-vascular microenvironment [5]. Microglia are sensitive to minute perturbations in homeostasis, necessary for fine tuning the local milieu yet potent purveyors of pathology should their activity become unbalanced. Impairment of microglia’s innate autophagic capacity can either lead to or accelerate neurodegenerative progression, as can an overabundance of microglial-derived proinflammatory signaling following infiltration of the blood-brain-barrier by peripheral immune cells [6]. Here we discuss the role of microglial interactions with the extracellular matrix (ECM) in both maintaining homeostasis during health and driving pathogenic changes with aging or disease. The ECM is not an inert extracellular substance, but a dynamic landscape with important functions in the CNS. We explore how microglia may interact with the ECM to facilitate their integral functions.

## Main text

### Microglia: helping to shape the central nervous system milieu

Microglia’s active role in CNS neuronal, vascular, and ECM remodeling spans development, when they are lowest in number but the most mobile, to injury, disease, and senescence [7, 8]. Microglia are neither bone marrow-derived nor replenished from the blood supply but rather are self-renewing through a tightly regulated cycle of spatial and temporal coupling of proliferation and apoptosis, which keeps their number relatively constant following development through normal maturation and aging, despite turning over several times during the lifetime [9]. Microglia are the workhorses of the CNS, active from early development through senescence, and can shift between metabolic programs (oxidative phosphorylation versus aerobic glycolysis) in response to stress and demand [10, 11]. Microglial functions, from development to disease, have been reviewed heavily and we refer the reader to Table 1 for a selection of recent literature.

Microglia help shape the neuronal and axonal milieu in the developing CNS [12]. For example, microglia directly impact developmental myelination of axons by phagocytosis of immature oligodendrocyte progenitor cells (OPCs), stopping their maturation and thereby regulating the formation of myelin [13]. At the other end of the life cycle, in the aging brain, where demyelination thins white matter tracts, activated microglia release factors including the hormone IGF-1 (insulin-like growth factor-1) that suppress OPC differentiation to impede remyelination [14]. During demyelination, microglia express genes relating to chemoattraction, complement, and phagocytosis, as in experimental autoimmune encephalomyelitis [15, 16]. Following demyelination, the ECM can become enriched with molecules like fibronectin and chondroitin sulfate proteoglycans (CSPGs), which actively inhibit the recruitment and differentiation of OPCs. During remyelination, microglia create an environment conducive to OPC responsiveness, secreting regenerative factors that help reshape the landscape of the local ECM [17]. Microglia release matrix metalloproteinases (MMPs), enzymes that break down these inhibitory molecules, as well as transglutaminases, creating a more permissive environment for OPCs to migrate into the lesion and mature into myelin-producing cells [18–20]. Microglial motility and phagocytosis are important not only in the development and maintenance of CNS neuronal connections, but for clearing cellular debris after tissue damage as well. By interacting with both astrocytes and pre- and post-synaptic neuronal membranes, microglia refine connections not only during CNS development [21–25], but in maintaining adult homeostasis [13, 26–28], and in neurodegenerative disease [6, 29–34].

Microglia are well-known for helping to mediate synaptogenesis during development. This process may be necessary for refinement of functional circuits; when microglial engulfment of synaptic proteins is altered, circuits form improperly [23, 24], though emerging evidence suggests otherwise [35]. Microglial-mediated synapse elimination is activity-dependent, with less active neurons preferentially targeted [27]. In their role as dynamic sculptors of neuronal circuits, microglia also modulate functional and structural synaptic plasticity in the adult brain and respond rapidly to changes in neuronal activity to help regulate circuits, including those in the hippocampus involved in learning and memory [36–39]. Homeostatic microglia dynamically interact with neuronal synapses to sense and regulate neural activity, such as in long-term potentiation and long-term depression, mechanisms underlying learning and memory [40]. Microglia modulate synaptic plasticity by physical interactions as well as through the release of soluble factors, including neurotrophic factors such as brain-derived neurotrophic factor (BDNF) [41, 42], complement factors [43], and interleukins such as IL-33 [41]. IL-33 instructs microglia to directly engulf components of the perisynaptic ECM to promote the formation of dendritic spines, which are crucial for synaptic plasticity and memory formation [41]. Microglia also contribute to memory consolidation by facilitating the stabilization of memory-associated synapses. However, excessive or abnormal microglial activity in this process can also lead to the elimination of synapses, potentially contributing to forgetting or memory loss such as that observed in Alzheimer’s disease [44]. The highly dynamic but persistent monitoring of the extracellular milieu by microglia uniquely positions them to respond to the rapid changes that occur in the synaptic microenvironment of neuronal networks [45].

The term ‘microglial pruning’ is typically used and suggests that the refining of synapses is a microglial-driven tagging and phagocytotic process; however, direct evidence of this particular process is scarce [46]. Two novel mechanisms of synapse elimination have been proposed and more effectively describe synaptic refinement by microglia: synaptic culling, whereby microglia migrate to directly sever the neuronal plasma membrane, and scavenging, whereby synaptic pruning is primarily neuronal driven, and microglia migrate to phagocytose the remaining debris [46]. In response to localized tissue injury, this process can involve both motility of microglial processes and frank migration of cell bodies [47]. Elimination of unwanted synapses during development versus cleanup of cellular debris following tissue damage represent independent phagocytic processes carried out by microglia [47, 48]. The complement system, part of the innate immune response, is important in recognizing and eliminating pathogens and damaged cells [49]. In the context of synaptic pruning, the complement factors C1q, and C3, and the integrin-associated protein CD47 which interacts with complement receptors such as SIRPα, are integral to process motility associated with synaptic refinement [50–53]. C1q and C3 bind to specific synapses, marking them for removal by microglia [54].

**Table 1:** Additional comprehensive reviews of microglial functions CNS during development, health homeostasis, and disease and degeneration

Focus	Reference	Title	Key Points
Development	*Barry-Carroll and Gomez-Nicola*,* 2024*	The molecular determinants of microglial developmental dynamics	- Migration of microglia progenitors takes place in the vasculature - Microglial distribution across the brain is influenced by regional levels of IL-34 and CSF1 - Compared to the adult brain, embryonic and postnatal microglia display a larger capacity to proliferate
	*Mehl et al.*,* 2022*	Microglia in brain development and regeneration	- Tissue microenvironments drive chromatin modifications that impact transcriptional regulation of microglial phenotype - Microglial heterogeneity is defined by CNS region and age - Microglial crosstalk with multiple cell types including neurons, oligodendrocyte precursors, astrocytes, and blood vessels highlights broad spectrum of roles in CNS homeostasis
Health and Homeostasis	*Kent and Miron*,* 2024*	Microglia regulation of central nervous system myelin health and regeneration	- Microglia undergo significant transcriptional changes during demyelination and remyelination - Microglia are required to regulate myelin during homeostasis - Aging, peripheral factors such as inflammation and exercise, and cellular interactions influence microglial actions during homeostasis
	*Pereira-Iglesias et al.*,* 2025*	Microglia as hunters or gatherers of brain synapses	- Proposes a new conceptual framework distinguishing between two potential mechanisms of synapse elimination by microglia: culling and scavenging
Disease and Degeneration	*Gao et al.*,* 2023*	Microglia in neurodegenerative diseases: mechanism and potential therapeutic targets	- Microglia exhibit both protective and detrimental roles in the CNS - Dysregulation of microglia may cause impaired phagocytosis of pathological deposits or their increased deposition, neuroinflammation, and microglial phenotype switching driving neurodegeneration - Dysfunctional microglia may also promote the clearance of synapses and ECM such as perineuronal nets - Microglial dysfunction is distinct across different neurodegenerative diseases
	*Bartels et al.*,* 2020*	Microglia modulate neurodegeneration in Alzheimer’s and Parkinson’s diseases	- Healthy communication between microglia and neurons is important in homeostasis; disruptions in this crosstalk can lead to persistent synaptic and neuronal dysfunction contributing to degeneration - Understanding and modulating interactions between microglia and neurons is crucial for the development of effective therapies

### The dynamic microglial cell

As resident macrophages, microglia interact with all other cell types in the CNS (including components of the microvasculature) in a highly dynamic and motile way that involves not only changes in location through migration, but in the morphology of individual cells [55]. Such changes are often looped into the broader concept of activation [56]. Long thought to exist in either of two distinct states, quiescent (or even dormant), in the homeostatic environment, but quickly transitioning to reactive in response to insult or disease, microglia are highly vigilant sentinels. In fact, microglia can occupy a range of states that reflect structural, functional, molecular, transcriptomic, and proteomic changes that can vary across brain regions and over time. These include rapid changes in morphology, gene expression, and physiology that reflects their constant surveillance of their immediate environment [57, 58]. Even the idea of quiescence in the context of microglia is a bit of misnomer, reflecting a specific neurochemical and transcriptional state that can best be described as what it is *not*, i.e., immune activated, but in no way implying lack of activity [57]. Quite the contrary, as microglia exist in a constant state of surveillance, polling their immediate microenvironment for factors that challenge normal physiological function: protein aggregates, antigens, microbes, unneeded or underutilized synapses, and of course, apoptotic or necrotic cells [59]. This role is both facilitated and characterized by a uniquely dynamic morphological phenotype, in which ramifying processes vacillate between protrusion and retraction over even large distances, with morphology and mobility that reflect the degree and quality of insult or stimulation [55, 59].

Microglia are well-tuned to sense an environment dominated by neuronal cells and their messenger molecules, extending their processes spontaneously during basal surveillance or through more directed motion in response to disease- or age-induced challenges to homeostasis [60, 61]. During the state of surveillance in normal tissue, microglial bodies and primary branches are quite stable, while higher-order ramified processes undergo rapid extension and retraction over seconds and minutes [62]. Microglia become increasingly mobile in disease or injury, as they sense and respond to a variety of molecular and mechanical signaling cues [63–66]. In response to the release of adenosine triphosphate (ATP) from focal trauma or tissue damage, microglia processes converge on the site of injury to envelop damaged tissue while retracting from the opposing direction; this reorganization can occur even without abject cell body migration [62], similar to synapse elimination [62, 67]. In contrast, in pathological conditions endemic to disease, microglia adopt an ameboid morphology to migrate over longer distances to phagocytize cellular debris [68]. This process is akin to macrophage phagocytosis during peripheral tissue damage [69]. In yet another process leading to engulfment of debris without cell body migration, microglia extend processes independently to form phagocytic compartments or pouches [68]. In this instance unoccupied microglial processes are able to continue surveillance of the surrounding tissue while engulfment and phagocytosis occur simultaneously in another compartment [68].

As microglia extend processes to monitor and respond to cues from nearby cells, they do not do so in a vacuum, but through interactions with large areas of ECM, the composition of which helps define by the local environmental niche [70, 71]. Such selective and directional tuning of morphological changes in microglial processes implies spatially (and temporally) coordinated manipulation of the ECM, which is a dynamic landscape under constant breakdown and reconstruction by cells, including microglia [72]. Typically, ECM remodeling is considered at the multi-cellular level, for spatially determined regions of whole tissue. For example, in development, ECM remodeling is necessary for cellular specification and organ morphogenesis, and mutations in ECM components cause widespread and often embryonically lethal tissue abnormalities [73]. As another example, in tumor metastasis, ECM remodeling in distant organs arises from biochemical modifications of tumor-associated ECM induced by stromal cells to support progression [74]. The changes exerted by and influencing the motility of individual microglial processes, by comparison, are extraordinarily focal, just a fraction of the small diameter of the diameter of the cell body (typically, 3–6 μm; [75]).

**Fig. 1:**
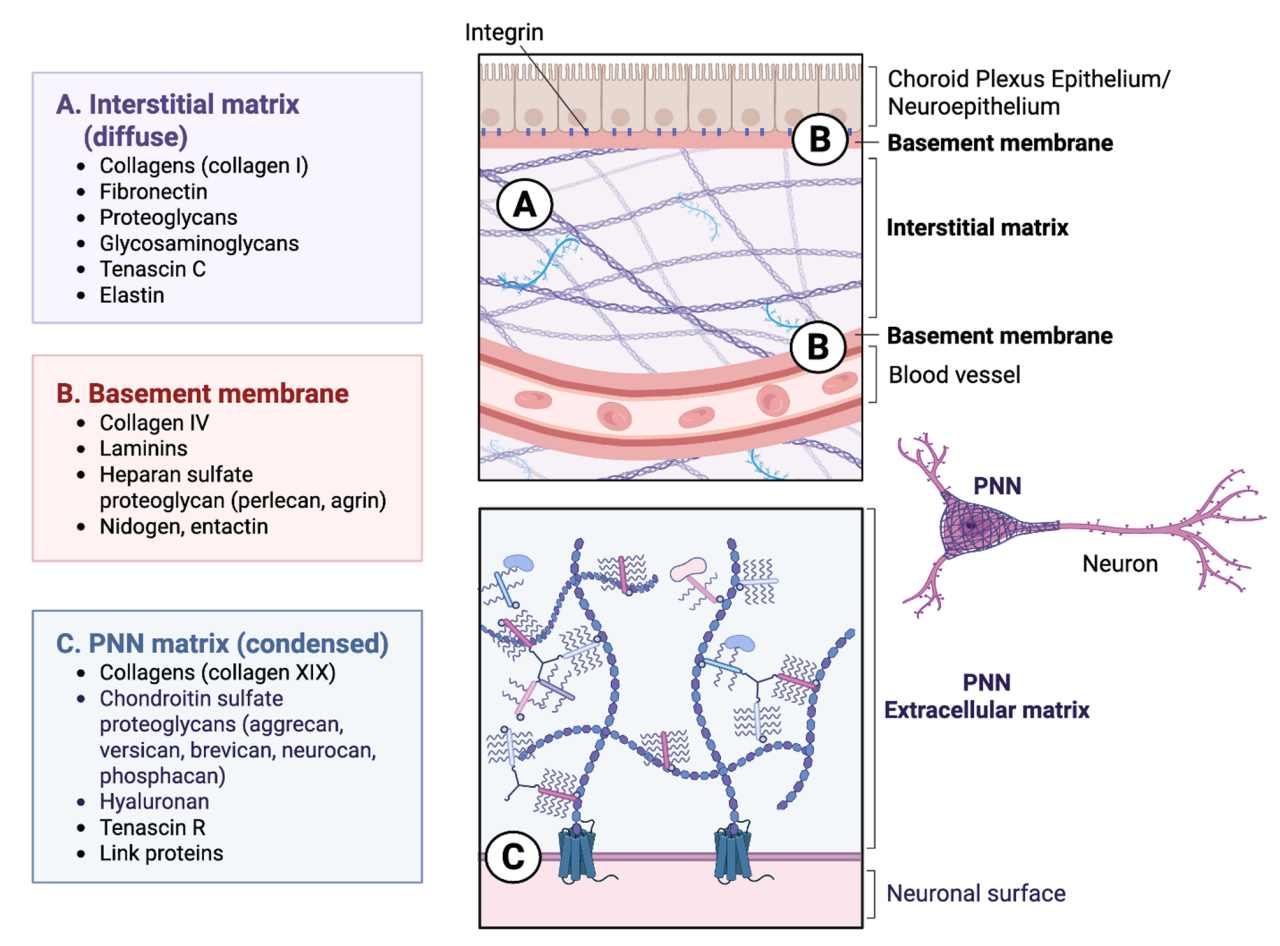
Structural and Molecular Organization of Brain ECM Compartments. Schematic highlighting the three major extracellular matrix domains in the brain. **(A)** The interstitial matrix (i.e., diffuse), composed of fibrillar collagens (e.g., collagen I), fibronectin, proteoglycans, glycosaminoglycans, tenascin-C, and elastin, provides structural support between cells. **(B)** Basement membranes, situated at neuroepithelial and vascular interfaces, contain collagen IV, laminins, heparan sulfate proteoglycans (e.g., perlecan, agrin), nidogen, and entactin, supporting barrier function and cellular anchorage. **(C)** Perineuronal nets (PNNs; i.e., condensed), which ensheath neurons, are enriched in collagen XIX, chondroitin sulfate proteoglycans (e.g., aggrecan, brevican), hyaluronan, tenascin-R, and link proteins, contributing to synaptic stability and plasticity. Integrin-mediated interactions anchor cells to ECM components, orchestrating signaling and structural integrity

**Fig. 2:**
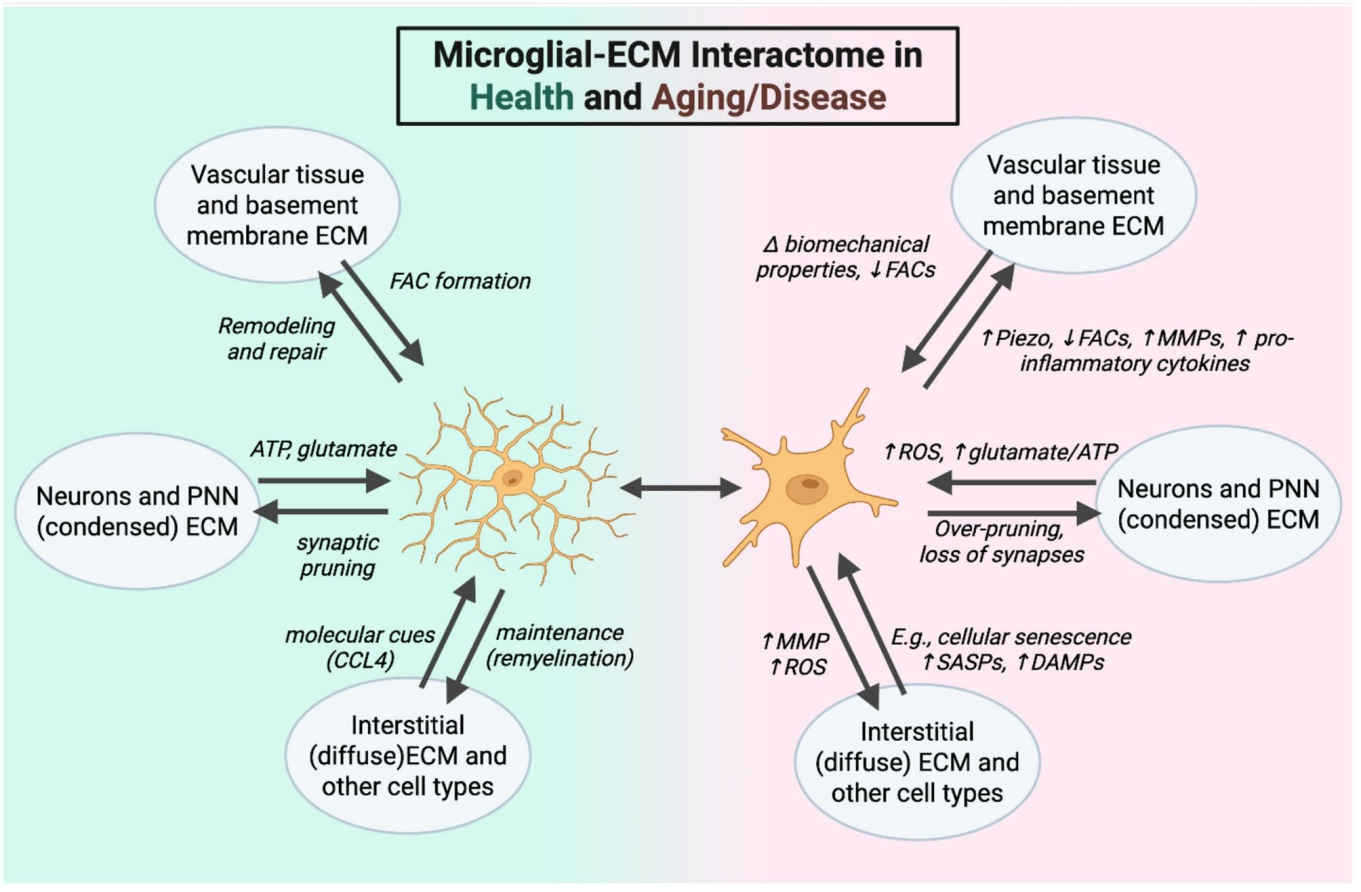
Microglial-ECM Interactome in Health and Aging/Disease. Schematic illustrating the bidirectional interactions between microglia and distinct ECM compartments—vascular, neuronal (including perineuronal nets), and interstitial ECM under homeostatic conditions (left) and during aging or disease (right). In health, microglia contribute to controlled ECM remodeling, synaptic pruning, and myelin maintenance via ATP, glutamate, and chemokines such as CCL4. In pathological states, altered mechanotransduction (e.g., Piezo activation), reduced formation of focal adhesion complexes (FACs) or expression of FAC components, leads to elevated MMPs, ROS, and pro-inflammatory cytokines disrupting ECM integrity. These events ultimately lead to synaptic loss, neuroinflammation, and impaired repair processes driving degeneration

The ECM, once considered an inert tissue scaffold, is now recognized as a biologically active substrate with roles in cell signaling, cell motility, and tissue infrastructure [76–78]. It is comprised of an array of proteins that include proteoglycans, laminins, tenascins, glycosaminoglycans, and of course collagen [72, 76]. In the brain, ECM can be partitioned into three structural types based on general organization and protein composition: diffuse interstitial and perisynaptic, condensed in perineuronal nets (PNNs), and basement membranes at the blood-brain barrier (BBB) and meninges and choroid plexus (Fig. 1) [79, 80]. Microglia are integral to both the development and maintenance of each ECM type, directly impacting the cells that reside within the ECM structures themselves in both health and disease [26, 29, 30, 41]. For example, consider PNNs, which are molecular scaffolds that stabilize and regulate mature synapses [72]. PNNs encapsulate the cell soma, dendrites, and axon initial segment and help to restrict synaptic plasticity, particularly in pathways exhibiting developmental activity-dependent plasticity such as the visual system [81]. PNNs are also implicated in the regulation of activity at tripartite synapses, which comprise an astrocyte process interacting with a presynaptic terminal and postsynaptic neuron [82]. In the homeostatic brain, basal regulation of PNN formation is mediated by microglial activity; PNNs dramatically increase in healthy adult brain after microglial depletion (Fig. 2) [26, 29, 30]. In contrast, degradation of the PNN by microglia that accompanies aging, cognitive decline, and neurodegenerative disease, is generally associated with increased microglial activation and secretion of ECM-degrading enzymes [83–87], which directly impacts the neurons and astrocytes within the PNN scaffold (Fig. 2).

The BBB is essential for maintenance of immune privilege in the CNS, forming a physical barrier that separates the parenchyma from the blood stream [88]. Together with endothelial cells, pericytes, astrocytes, and the collagenous basement membrane, microglia help form a selectively permeable membrane through their maintenance of BBB ECM [89, 90]. Microglia are key regulators of CNS vascular development and its maintenance [91]; many remain perivascular cells, acting as the first line of defense for the CNS [90]. Brain injuries such as those exhibited after electrode placement trigger microvascular rupture and BBB leakage, suggesting that immediate diffusion gradients of molecules, sugars, proteins, and other components from the blood into tissue trigger microglia motility over periods that stretch from a few hours to days [92, 93]. By responding to signaling molecules that are derived from vascular endothelial cells and are BBB-permeable (like the chemokine CCL5 or RANTES), microglial migration to damaged vessels first initiates increases in the tight junction protein claudin-5 in an apparent attempt to bolster the BBB, thereby protecting the CNS milieu (Fig. 2) [94]. However, as BBB permeability increases, depositing molecules foreign to the CNS, such as albumin, immunoglobulins (IgG/IgM/IgA), fibrinogen, complement, and red blood cells, into the neuronal milieu, microglial migration is accelerated [94–97]. Microglia quickly switch course to releasing pro-inflammatory molecules and degradative enzymes targeted to the ECM that in turn breaks down astrocyte end feet connections and further degrades BBB integrity [98].

### Drivers of microglial motility in the matrix

The carefully orchestrated movements that microglia execute allow interaction with synapses, neurons, and of course astrocyte glia in response to a variety of molecular cues that vary with the type of stress induced [50, 99]. There are a variety of environmental stimuli that microglia may respond to for facilitating motility (Table 2) [100]. These stimuli include diffusible chemical cues such as cytokines and growth factors (chemotaxis; [101]), cues bound to a substrate such as the ECM such as the Rho family of small GTP-binding proteins (haptotaxis; [102]), differential ECM compliance such as changes in stiffness of the ECM or stiffness gradients (durotaxis; [103]), geometric properties of a migration substrate such as alignment of collagen fibers (topotaxis; [104]), and ionic differences generated by biological barriers such as an electrical potential gradient (galvanotaxis; [105]). Since microglia are motile in different ways, they may utilize multiple environmental cues -especially in comparing whole cell migration vs. extension and contraction of processes.

Migration towards (or away) from a variety of environmental cues is integral to many biological processes, including in wound healing by fibroblasts and epithelial cells [106], neutrophil migration [107], including migration by microglia [108]. Cell migration is a highly coordinated and tightly controlled process requiring changes in cell morphology and interactions with the ECM [109]. Microglia must be able to not only sense or detect initiating cues that induce mobility but also interact with and restructure the ECM to promote migration of their cell bodies and/or reorganization of their processes [108]. Microglia respond to the dynamic CNS environment through the expression of a rich variety of cell surface receptors, which collectively comprise the microglial “sensome” [4, 110]. Migration, which is a form of directed motility, depends upon focal adhesion complexes. These are integrin-containing protein structures that help anchor intracellular filaments in cells (including microglia) to the ECM [111]. Migration is executed through a cycle that can be broken down into four discreet steps: 1) protrusion of the leading edge of the cell, 2) cell adhesion to the ECM, 3) generation of traction stresses against adhesion complexes, and finally, 4) release of rear adhesions and cell body contraction [112–114]. The speed and direction at which cells migrate is modulated through adhesion complex formation and the density and mechanical compliance of the ECM, a process that may involve integration with the filamentous actin (F-actin) cytoskeleton, protein tyrosine phosphorylation and myosin-generated forces [113, 115–117].

For migration to occur, microglia must exert forces on their environment, which requires sensing mechanical properties like tissue stiffness, which is determined in large part by ECM. Increased stiffness enhances both microglial morphological complexity and their release of inflammatory mediators; in vitro, microglia migrate towards stiffer substrates [118]. Mechanosensitive receptors enable microglia to detect alterations in the biomechanical properties of surrounding cells and tissues [119–122]. For example, the expression of PIEZO1 and TRPV4, both mechanosensitive cation channels, modulate microglia migration and release of pro-inflammatory cytokines by sensing gradients in tissue stiffness through a Ca^2+^-dependent process [120, 121, 123].

Microglial mechanosensation is also important in disease. Vascular calcifications comprise one of the pathological hallmarks of a wide range of diseases triggering microglial migration and inflammation [124]. It is possible that microglial function is critical to vascular calcification, as they appear to sense and degrade calcifications, possibly through changes in biomechanics of vessel tissue [124]. In Alzheimer’s and Parkinson’s, excessive extracellular accumulation of proteins such as Aβ, hyperphosphorylated tau, and α-synuclein occur before significant cognitive decline [125]. During progression of Alzheimer’s, Aβ forms aggregates as its structure changes, evolving from amyloid fibrils to formation of insoluble plaques [4]. Although not typically regarded as ECM proteins, accumulations of Aβ and tau in the extracellular space interact with ECM as a substrate, thereby altering the structure of the ECM itself [126–130]. With extracellular accumulation, alterations occur in the biomechanical forces between protein, ECM, and points of cell-surface contact. The role of microglia in detecting amyloid and aiding in its clearance is conflicting, with emerging evidence suggesting microglia are fundamental to reshaping amyloid plaques later in the disease [131, 132]. The distinct physical features of amyloid plaques include high rigidity, which may trigger microglial mechanosensing of the plaque as a foreign body, thus promoting migration and activation [133]. This migration likely occurs through PIEZO1 channels [134], possibly directed toward a chemotactic signal formed by apolipoprotein E (ApoE) associated with amyloid plaques and the cell surface adhesion molecule VCAM1 [135–138].

Microglial motility through the ECM very likely involves sensing local cellular signaling and neuronal activity. Receptors expressed by microglia detect changes in chemokines and cytokines, purinergic molecules, inorganic substances, as well as changes in pH and amino acids [110, 139]. For example, in experimental autoimmune encephalomyelitis (EAE), astrocytes demonstrating a proinflammatory phenotype are influenced directly by chemokine-mediated signaling in microglia [140]. Blood-derived signals can influence both microglia and astrocytes through commonly expressed receptors, such as the aryl hydrocarbon receptor, while microglia and astrocytes influence the inflammatory status of the other through release of cytokines such as TNFα (tumor necrosis factor) and interleukins [141].

In glioblastoma, an aggressive form of astrocytic cancer, microglia are the most abundant population of primary immune cells, accounting for up to 50% of total glioblastoma tumor mass [142]. Microglial migration and infiltration in glioblastoma is driven by glioma expression of the chemoattractant CCL2 [143]. A key mechanism driving microglial invasion in glioblastoma are changes in cellular contractility and biomechanics [144]. Microglial migration in glioblastoma depends heavily on binding to fibrous proteins in the ECM including glycosaminoglycans (GAGs), chondroitin sulfate proteoglycans (CSPGs), and laminin, as well as extracellular Ca^2+^ and glutamate [145]. A high concentration of extracellular glutamate can lead to degeneration of neurons, carving a path through the ECM for glioblastoma cells to migrate [146–148]. Interestingly, in vitro microglial cells exhibit strong migratory responses to extracellular glutamate [149]. Could neuronal release of glutamate in a dysregulated fashion play a mechanistic role in CNS microglial migration? Possibly. Accumulation of glutamate in the neuronal synaptic cleft has long been argued a driving force of neurodegeneration in age-related CNS diseases [150]. Such accumulations (e.g., due to neuronal hyperexcitation, uptake and transport dysfunction, or release of glutamate from astrocytes exposed to Aβ or α-synuclein oligomers) may trigger migration of microglia or motility of their processes.

Microglia express additional mechanisms that likely contribute to both motility and remodeling of their processes in the ECM. In homeostasis and disease, neuronal excitation itself alters neuron-microglia interactions by affecting microglial process extension and motility [151–153]. Like neurons and astrocytes (and, to a lesser degree, oligodendrocytes), microglia express a variety of ion channels that contribute to chemotaxis along concentration gradients, migration, phagocytosis, and inflammatory signaling [154, 155]. In basal conditions, microglia respond to neuronal activity by sensing neurotransmitter and extracellular ions, leading to changes in their resting membrane potential [139]. These signals include changes in concentration of ATP and glutamate [139, 149, 156, 157]. In fact, ATP is one of the most characterized microglial motility and chemotactic cues; it is released by neurons, microglia and other cells in the CNS [158, 159]. The detection of extracellular ATP and subsequent chemotaxis are mediated by the purinergic G_i_-protein-coupled receptor P2Y12 and ATP-gated ion channel receptors P2X4 and P2X7 [62, 67, 160, 161].

Microglial migration is also impacted by biomechanical changes to tissue. In glaucoma, a disease that causes degeneration of the optic nerve, forces exerted by intraocular pressure at the optic nerve head as it adjoins the eye alter its biomechanical properties in part through changes in ECM deposition [162–164]. Coincidentally or not, microglia are often the “first responders” at the nerve head in glaucoma. Their activation and migration to this critical location may be driven in part by mechanosensitive changes in the ECM that promote chemotaxis to the area of stress through microglial expression of P2X4 and P2X7 receptors [165, 166]. In a mouse model of retinal degeneration, microglial phenotype and the control of vascular architecture depends on tissue stiffness and is regulated by signaling through integrins, which are ECM receptor binding proteins [167].

Gradients of migratory molecules in the ECM and alterations in tissue biomechanics both play a role in the initiation and speed of microglial cell migration. Each of these migratory triggers is in some way linked with the ECM. The ECM binds to a variety of molecules, including growth factors and chemokines, creating concentration gradients that guide cellular behavior [168]. The structure and composition of this matrix can influence how effectively these molecules diffuse and how accessible they are to microglial receptors [168]. Specific ECM components, such as proteoglycans and polysaccharides, determine the size of the extracellular space and thus regulate the diffusion of molecules [169]. For instance, a high concentration of negatively charged CSPGs can create a dense matrix that hinders the movement of molecules and migration of cells [170]. Similarly, the high negative charge of PNNs allows them to bind ions and signaling molecules such as growth factors [171].

Inflammatory mediators such as chemokines, cytokines, and reactive oxygen species can be sequestered by binding to the ECM and contribute to overall directed motility and chemotaxis of microglia towards amyloid deposits in Alzheimer’s, Parkinson’s, and multiple sclerosis [172]. Microglia sense and respond to paracrine signals like ligands or other soluble molecules but also have the capacity to function in an autocrine fashion [158, 173, 174]. Important receptor axes specifically involved in chemotaxis of microglia include P2Y, TREM2, CD33, IL-3/IL-3R, IL-33/ST2, CCL2/CCR2, CX3CR1, CCL5, and integrins [4, 94, 159]^175^– [177]. Interestingly, low intensity pulsed ultrasound stimulation increases the velocity at which microglia travel toward electrode-induced injury; an effect thought to be mediated by activation of mechanosensitive channels, or amplified release of cellular components known to increase migration such as ATP [93].

**Table 2:** Overview of key signaling pathways regulating microglial motility, including environmental cues, mechanosensitive receptors, adhesion molecules, and chemotactic signals involved in both homeostasis and disease

Category	Key Pathways/Molecules	Function in Microglial Motility
Mechanosensation	PIEZO1 channels	- Microglia sense changes in tissue stiffness → Ca²⁺ influx → modulates migration and cytokine release
Focal Adhesion and ECM	Integrins, ECM components (e.g., CSPGs, Laminin)	- Anchors microglia to ECM, helping to transmits mechanical forces, enables traction of microglia for movement
Purinergic Signaling	P2Y12, P2 × 4, P2 × 7	- Detect extracellular ATP → guides directed movement
Cytokine & Chemokine Receptors	TREM2, CD33, CX3CR1, CCL2/CCR2, CCL5, IL-3R, IL-33/ST2	- Mediate chemotaxis, especially in inflammation and disease
Neurotransmitter Signaling	Glutamate, ATP	- Drives migration and process motility via synaptic and injury signals - Microglia exhibit motility responses to extracellular glutamate, especially in neurodegenerative disease contexts - Ca^2+^ signaling through ion channels (often in response to neurotransmitters or mechanical cues) influences process extension and migration

### The microglia-ECM “interactome” and neurodegenerative disease

The ECM is a central player in the interplay between extracellular signaling and microglial migration and motility [41, 72, 73, 79, 108, 109, 178]. The ECM in the healthy CNS undergoes dynamic turnover through a highly regulated system of proteases and their inhibitors [179]. Turnover is important in many homeostatic functions of the CNS, including synaptic remodeling, which is in part mediated by microglial phagocytosis of ECM that is triggered by neuronal-derived interleukin 33 (IL-33; [180]). In disease, however, regulation of ECM turnover is disrupted, in part due to the activity of microglia [179]. ECM degradation in neural tissue is accompanied by high levels of extracellular proteases, such as matrix metalloproteinases (MMPs), and degradation and remodeling of the perivascular and perineuronal ECM [179]. Such actions lead to over-pruning of neuronal synapses and increased inflammation associated with neurodegeneration [181, 182].

After sensing environmental cues to migrate, microglia initiate contact with their surroundings, primarily through the expression of integrin receptors. The type of contact microglia make with ECM components is influenced by extracellular cytokines such as TNFα and the immune-derived interferons IFN-α and IFN-γ, which in turn upregulate the expression of ECM receptor binding proteins including integrins [183–185]. Upregulation of integrins promotes the interaction between microglia and the ECM through the formation of the focal adhesion complex [185]. The ECM itself functions as a medium for the sequestration of not only cell signaling molecules such as glutamate, but also divalent metal ions such as calcium (Ca^2+^), zinc (Zn^2+^), and magnesium (Mg^2+^) [186]. In areas outside of the CNS divalent metals directly impact cancer cell migration by acting as gradient-forming agents, associating with GAGs, and enhancing integrin activation in the ECM [187]. In vascular endothelial cells and neurons, manganese (Mn^2+^) acts as a potent integrin activator, inducing the formation of focal adhesion complexes [188, 189].

Interestingly, microglia release proteases and phagocytize proteins in a feed-forward cascade of tissue-damaging events that accelerate ECM restructuring. Such alterations can include changes in ECM biomechanics that directly impact microglial function, which in turn may promote excessive ECM degradation and eventually impact neuronal survival. For example, altering the ECM impedes microglial-mediated synaptic remodeling by preventing the deposition of complement proteins, leading to impaired expression of the microglial sensome [172, 190]. In multiple sclerosis, lesions of demyelination also contain large numbers of microglial cells linked to ECM dysregulation [191]. In glioblastoma, microglia may drive ECM changes that promote tumor growth and invasion. Microglia migrate to and invade glioblastoma masses, in turn modifying ECM composition through MMPs and cathepsins [76, 144, 192]. These enzymes produce fragments of ECM proteins with new biological activities (called “matrikins”) that influence glioma cell behavior [192]. High levels of ECM proteins including collagen I, collagen IV, laminin and fibronectin are routinely found in glioblastoma tissue, and elevated collagen IV correlates with poorer prognosis [193].

Just as the sensome comprises the collective of cell-surface receptors through which microglia detect changes in the CNS microenvironment [110], we propose the “interactome” as the molecular machinery microglia employ to interact with and manipulate the ECM. If the sensome is the “afferent” system microglia employ to gather information about their immediate environment, the interactome is the “efferent” system through which they act upon their environment. Through the interactome, microglia can create small modifications of the ECM necessary for homeostatic surveillance and more dramatic adaptive behaviors necessitated by challenges to homeostasis. The interactome is dependent on the environmental niche in which microglia reside; for example, vascular basement membrane-associated microglia will have an ECM-interactome that is different to microglia associated with PNNs at neuronal synapses. The interactome is dynamic, influenced by changes in surrounding cells and molecular signals, dictating how microglia respond to changes with aging and in disease.

The cellular consequences of the interactome are vast in homeostasis and disease. In healthy conditions, the interactome of microglia within their various spatial niches is balanced, enabling microglia to carry out their executive functions in a controlled manner (Fig. 2). However, even slight changes in ECM composition and structure have downstream effects on the biomechanical properties of the matrix, the formation of focal adhesion complexes, release of matrikins from the ECM, and the exposure of binding sites and chemokines for cell motility and inflammatory signaling [76, 178, 194]. Such changes in the microglial-ECM interactome trigger an imbalance and can ultimately promote a cascade of pro-degenerative events. For example, an imbalance between collagen and elastin in the ECM leads to stiffening of vascular tissue with age, the cause of which is unknown [195]. The downstream effects of tissue stiffening on the interactome can be hypothetically mapped out: vascular stiffening alters the biomechanical properties of the tissue sensed by microglia through PIEZO channels [196]. Microglial stiffness sensing leads to a downstream cascade of pro-inflammatory cytokine and MMP release, leading to increased degradation of the ECM in a feed-forward cycle (Fig. 2). Such degradation of the ECM promotes the release of bioactive matrikins and also creates space within the ECM for microglial migration.

There is growing evidence of brain ECM changes in human disease; cerebrospinal levels of MMPs-2 and − 7 are associated with brain amyloid deposition and the severity of white matter lesions [197]. Similarly, microglia in close proximity to neurons maintain synapses in a process of culling or scavenging during homeostatic conditions. However, with aging and in disease, dysfunctional neuronal activity can lead to accumulations of excess glutamate and increased reactive oxygen species (ROS). Glutamate receptors on microglia (e.g., AMPA receptors) accumulate at focal adhesion sites where they may indirectly mediate interactions between the ECM and integrins [198]. Such changes in the ECM interactome of microglia triggers the upregulated release of MMPs and other proteases which directly impact the local ECM milieu facilitating microglial motility [199] and increased (i.e., uncontrolled) synaptic pruning. Other cell types within the CNS have the capacity to alter the ECM environment, for example, astrocytes are a primary producer of many ECM proteins including glycoproteins and collagen [76, 200]. Changes in astrocyte-derived ECM composition can also trigger so called “activation of microglia”, potentiating degenerative processes.

A hallmark of many neurodegenerative diseases is the patterned spread of degeneration, either confined within a specific brain region or disseminating across multiple areas over time. For example, in glaucoma, degeneration progresses in an arcuate-like pattern over time, mimicking the spatial profile of the retinal vasculature and axon bundles in the retina [201]. These characteristic degenerative patterns are common across patients, yet the mechanisms driving these spatially distinct patterns are elusive.

Microglia interact with neurons, astrocytes, and oligodendrocytes; astrocytes form interconnected networks via gap junctions, while neurons couple to one another and to astrocytes [202]. As might be gleaned from their broad distribution across the brain, microglia are a heterogeneous community with subpopulations based on specific brain regions; each population possesses unique transcriptional and functional features [58, 203]. However, despite this heterogeneity, alterations in the microglial-ECM interactome could have far-reaching consequences. If microglia communicate over larger distances, such as is evident in astrocytes of the optic nerve [204], local changes could propagate via interactions with the ECM, thereby creating a domino effect and spreading dysregulation across brain regions.

## Conclusions

Microglia represent a morphologically complex and heterogeneous community with subpopulations that vary across specific brain regions. This complexity is reflected in their broad functional repertoire, which includes not only critical roles in immune surveillance and response to insult or stress, but also in developmental synaptogenesis and in modulating synaptic plasticity in the adult brain in response to changes in neuronal activity. Microglia are self-renewing through spatial and temporal coupling of proliferation and apoptosis, which keeps their number relatively constant through normal maturation and aging. As the innate immune cells of the CNS, microglia are not only behind the blood-brain barrier (BBB) where they aid in detection of injury, cellular debris, and invasive pathogens, but aid in its development and maintenance along with endothelial cells, pericytes, and astrocytes [89, 90]. Microglia are also important regulators of vascular health [91], with many remaining perivascular cells throughout adulthood [90].

Microglia constantly survey their microenvironment for challenges to normal physiological function [55, 59]. In this role, microglia are highly sensitive to even minute perturbations in homeostasis, which requires complex interactions with the ECM. These include dynamic morphological reorganization with carefully orchestrated movements of their processes to support interactions with synapses, neurons, and astrocytes in response to molecular stress cues [50, 99]. This reorganization implies coordinated manipulation of the ECM by microglia [72], to promote migration of their cell bodies and/or reorganization of their processes [108]. This in turn involves sensing mechanical ECM-mediated properties like tissue stiffness, which influences both microglial complexity and capacity to detect additional alterations of tissues [119–122]. Microglia express a variety of ion channels that contribute to chemotaxis along concentration gradients, migration, phagocytosis, and inflammatory signaling [154, 155]. Gradients of migratory molecules that bind to the ECM help determine the speed of microglia migration, including growth factors and chemokines [168].

The ECM in the CNS undergoes remodeling through a finely regulated system of proteases and their inhibitors [179], which is in part mediated by microglial phagocytosis. In disease, however, regulation of ECM turnover is disrupted, in part due to the activity of microglia [179], which release proteases and phagocytize proteins to accelerate ECM restructuring. Such alterations directly impact microglial function, through a variety of cell-surface receptors [110]. This highlights the potential impact of the ECM-interactome on neurodegenerative disease progression. Perturbations in this interactome - whether due to aging or pathology - can initiate a cascade of maladaptive changes to the ECM in the brain. For example, an initial insult (such as increased amyloid deposition, as in Alzheimer’s disease) trigger changes in local tissue biomechanics, initiating changes in the microglial interactome causing excessive MMP release and ECM degradation. These changes, in turn, may further disrupt neighboring microglia interactomes, creating a spatially perpetuating cycle that ultimately leads to neuronal dysfunction and degeneration.

This raises several pressing questions: do microglia secrete specific signaling molecules to transmit interactome changes across their network? Are microglial responses primarily governed by ECM dynamics, or modulated through interactions with other glia and neuronal cell types? Do changes in tissue biomechanics, including ECM biomechanics, alter microglial migration? And critically, are cellular changes to microglia that are mediated by the interactome reversible? A key unresolved question is how the extracellular milieu impacts microglial communication as a coordinated, functional network.

Answering these and similar questions will require experimental approaches using complementary in vivo and in vitro systems. For example, hydrogel-based ECM stiffness assays, microglia-astrocyte-neuron co-cultures, and microfluidic devices may allow precise manipulation of cell-cell and cell-matrix interactions to discern local versus long-range signaling mechanisms [205]. Whereas in vivo models, in which ECM composition and tissue biomechanics are altered in defined regions, will enable the study of microglial responses and cell migration within intact neural circuits. Combining functional assays with ECM proteomics will help to identify molecular mediators of ECM-driven microglial dynamics.

In conclusion, we propose the microglial-ECM interactome as an emergent critical regulator of brain homeostasis and disease progression. The microglial-ECM interactome is not a singular mechanism for microglial activation and motility; it likely contributes to disease progression with other key modulators of microglial activity such as genetic alterations and neuroimmune modulation [206]. The interactome’s dynamic nature could allow for microglia to adapt to changes in the extracellular environment, facilitating cellular and process motility and other homeostatic microglial functions, however, this same responsiveness can also lead to maladaptive feedback loops in aging and neurodegeneration. The potential for microglia to operate as a coordinated network via the interactome introduces a new dimension to our understanding of neuroinflammatory spread and region-specific vulnerability. Elucidating the mechanisms that govern microglial communication and involvement of the ECM-interactome in disease will be essential for identifying novel therapeutic strategies aimed at halting or reversing the progression of neurodegenerative diseases. Strategies that attempt to establish clear mechanisms most usefully will meet strident conditions for causality. This includes transgenic approaches in which identification and modulation of a microglia-specific transcript change the composition or physical properties of the ECM in ways that clearly influence CNS tissues challenged by disease or stress.

## Data Availability

No datasets were generated or analysed during the current study.

## Abbreviations

ATP: Adenosine triphosphate
BBB: Blood–brain barrier
CNS: Central nervous system
CSPG: Chondroitin sulfate proteoglycan
DAMP: Danger–associated molecular pattern
EAE: Autoimmune encephalomyelitis
ECM: Extracellular matrix
GAG: Glycosaminoglycan
IGF: 1–Insulin–like growth factor–1
IL: Interleukin
MMP: Matrix metalloprotease
OPC: Oligodendrocyte progenitor cell
PNN: Perineuronal net
